# Secondary Syphilis Masquerading as Systemic Lupus Erythematosus: A Clinicopathologic Diagnostic Challenge

**DOI:** 10.7759/cureus.115015

**Published:** 2026-08-23

**Authors:** Reem AlQusaimi, Fawziah AlRujaib, Fahad AlSharhan, Fatma AlAmiri, Abdullah AlShatti, Bader Alflaij, Munirah F AlEnezi, Dana Alobaid, Qutov Alkhaldi, Sally Mohamed, Yousef Almutairi

**Affiliations:** 1 Dermatology, Abdulkareem AlSaeed Dermatology Center, Kuwait City, KWT; 2 Medicine and Surgery, Amiri Hospital, Kuwait City, KWT; 3 Medicine, Sheikh Jaber Al-Ahmad Al-Sabah Hospital, Kuwait City, KWT; 4 Dermatology, Al-Jahra Hospital, Kuwait City, KWT; 5 Medicine, Kuwait University, Kuwait City, KWT; 6 General Surgery, Farwaniya Hospital, Kuwait City, KWT; 7 Rheumatology, Amiri Hospital, Kuwait City, KWT

**Keywords:** acute cutaneous lupus erythematosus, clinicopathologic correlation, secondary syphilis, systemic lupus erythematosus, treponema pallidum

## Abstract

Secondary syphilis is known as the “Great Imitator” because of its diverse clinical manifestations, which may resemble infectious, inflammatory, and autoimmune diseases. Although it may mimic systemic lupus erythematosus (SLE), simultaneous clinical, serologic, and histopathologic features strongly suggestive of SLE are uncommon and can result in diagnostic error.

We report the case of a 51-year-old man who presented with a three-month history of bilateral conjunctival injection, symmetrical polyarthralgia, diffuse alopecia, oral ulceration, and a generalized pruritic erythematous maculopapular eruption. Initial investigations revealed positive antinuclear antibodies (ANAs), hypocomplementemia, elevated inflammatory markers, and a skin biopsy favoring acute cutaneous lupus erythematosus (ACLE), leading to a provisional diagnosis of SLE. Despite treatment with hydroxychloroquine, methotrexate, and corticosteroids, his cutaneous disease persisted. Subsequent testing demonstrated strongly reactive rapid plasma reagin (RPR) and *Treponema pallidum* hemagglutination assay (TPHA) results. Correlation of the clinical, serologic, and histopathologic findings established secondary syphilis as the underlying diagnosis.

This case illustrates that secondary syphilis can closely mimic SLE across multiple diagnostic domains. Recognition of this uncommon presentation emphasizes the importance of maintaining a broad differential diagnosis and integrating clinical, serologic, and histopathologic findings to avoid misdiagnosis and unnecessary immunosuppressive therapy.

## Introduction

Syphilis is a sexually transmitted infection (STI) caused by *Treponema pallidum* subspecies *pallidum* and remains a significant public health concern worldwide despite the availability of highly effective antimicrobial therapy. Secondary syphilis, the disseminated stage of infection, is characterized by a wide spectrum of mucocutaneous and systemic manifestations that frequently resemble other infectious, inflammatory, and autoimmune disorders, earning it the designation of the “Great Imitator.” Although its classic presentation includes a generalized papulosquamous eruption with palmoplantar involvement, numerous atypical clinical manifestations have been described, often resulting in delayed diagnosis or inappropriate management [1].

Systemic lupus erythematosus (SLE) is among the autoimmune conditions most commonly reported to overlap clinically with secondary syphilis. Shared features - including cutaneous eruptions, oral ulceration, alopecia, inflammatory arthralgia, constitutional symptoms, and positive autoimmune serologic markers - may obscure the underlying diagnosis and present a significant diagnostic challenge [2].

Although isolated cases have been reported, secondary syphilis presenting with concurrent clinical, serologic, and histopathologic findings strongly suggestive of SLE remains uncommon. We report a case of secondary syphilis that closely mimicked SLE, initially leading to an autoimmune diagnosis before clinicopathologic correlation and syphilis serology established the correct diagnosis.

## Case presentation

A 51-year-old man with no known history of autoimmune disease was referred to the rheumatology clinic with a three-month history of bilateral conjunctival injection, initially diagnosed by ophthalmology as viral conjunctivitis, accompanied by symmetrical polyarthralgia involving both elbows, wrists, hands, and ankles. He also reported morning stiffness lasting approximately 15 minutes, a generalized pruritic erythematous maculopapular eruption predominantly involving the trunk and upper extremities, progressive diffuse alopecia, and a painless ulcer over the hard palate. Regarding his epidemiologic and STI history, the patient denied recent sexual exposures or risk factors. He denied a preceding genital or extragenital chancre or other genital lesions and had no known history of previous syphilis testing or treatment. No previous STIs were reported or documented, and prior HIV testing had not been performed. He denied neurologic or otologic symptoms. Physical examination demonstrated conjunctival injection, diffuse erythema of the hard palate with a painless ulcer (Figure 1), and a generalized faint erythematous maculopapular eruption involving the trunk and upper extremities (Figure 2). The eruption spared the palms and soles, and no lymphadenopathy was identified.

**Figure 1:**
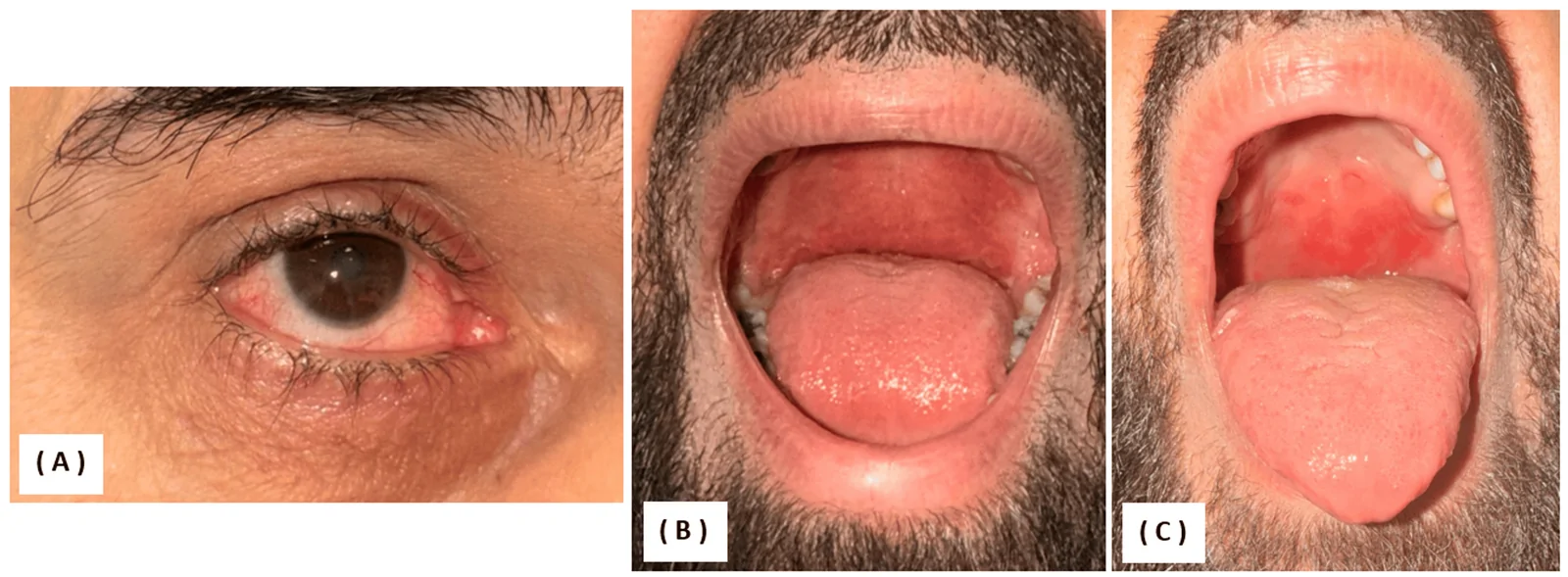
Clinical Photographs Demonstrating Ocular and Oral Mucosal Manifestations at Initial Presentation (A) Conjunctival injection of the left eye; (B) Diffuse erythema involving the hard palate; (C) Close-up view demonstrating a painless ulcer on the hard palate surrounded by erythematous mucosa.

**Figure 2:**
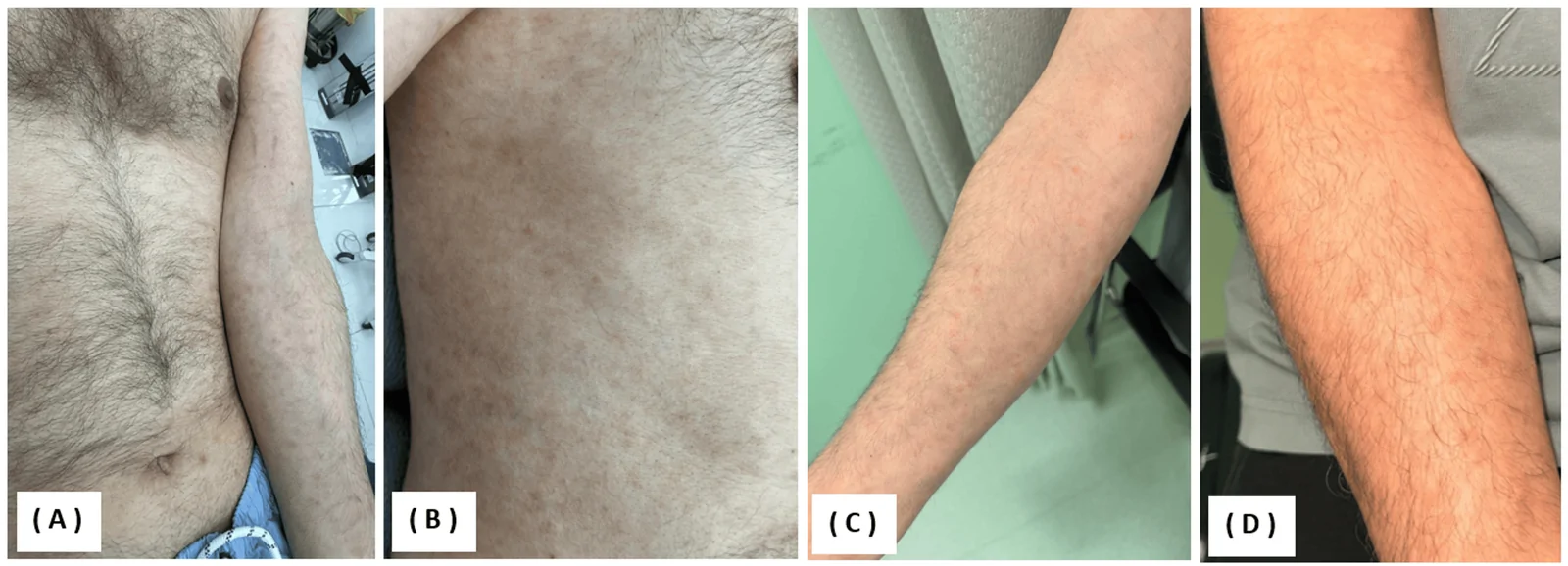
Clinical Photographs Demonstrating Generalized Cutaneous Manifestations at Initial Presentation (A) Diffuse eruption involving the anterior trunk and upper extremity; (B) Closer view of the trunk showing multiple faint erythematous macules and papules; (C) Multiple erythematous papules distributed over the forearm; (D) Higher-magnification view of the forearm demonstrating discrete erythematous papules.

Initial investigations demonstrated a positive antinuclear antibody (ANA) titer of 1:160 with negative anti-double-stranded DNA antibodies, weakly positive anti-Mi-2 antibodies, positive anti-GP210 antibodies, reduced complement C4 levels, an elevated erythrocyte sedimentation rate (ESR) of 97 mm/hour, and a C-reactive protein (CRP) level of 9 mg/L. Complete blood count and urinalysis were unremarkable, while serum angiotensin-converting enzyme (ACE) was mildly elevated at 57 U/L (Table 1). Chest radiography showed no abnormalities. Based on the clinical presentation and laboratory findings, SLE, Behçet disease, and sarcoidosis were considered in the differential diagnosis, with SLE regarded as the leading diagnosis. Hydroxychloroquine (200 mg and 400 mg on alternate days) was initiated, and the patient was referred to a combined rheumatology-dermatology clinic for further evaluation.

**Table 1:** Initial Laboratory Investigations Initial laboratory investigations performed during the diagnostic evaluation included inflammatory markers, serum ACE level, and autoimmune serology. ANA: antinuclear antibody; anti-dsDNA: anti-double-stranded DNA antibody; ACE: angiotensin-converting enzyme; ESR: erythrocyte sedimentation rate; CRP: C-reactive protein.

Investigation	Result	Reference range (Unit)
Inflammatory Markers		
ESR	97	0 - 15 (mm/hr)
CRP	9	0 - 8 (mg/L)
Serum Angiotensin-Converting Enzyme		
ACE	57	8 - 52 (U/L)
Autoimmune Serology		
ANA	Positive (1:160, homogeneous pattern)	-
Anti-dsDNA	Negative	-
Anti-Mi-2	Weakly Positive	-
Anti-GP210	Positive	-

At follow-up, the patient reported persistent cutaneous lesions involving the arms and neck, ongoing bilateral ocular redness primarily attributed to dry eyes, and worsening alopecia. His joint pain had improved after initiation of hydroxychloroquine. He also described two days of night sweats and an unintentional 5-kg weight loss over the preceding months. No significant new laboratory abnormalities were identified. Hydroxychloroquine therapy was continued while awaiting further dermatologic assessment.

During subsequent review, the patient continued to have a persistent non-blanching maculopapular eruption despite improvement in his articular symptoms. Methotrexate 10 mg weekly with folic acid supplementation was commenced, and a skin punch biopsy was arranged to clarify the underlying dermatologic diagnosis.

At review approximately three weeks later, despite receiving two doses of methotrexate in addition to hydroxychloroquine, the patient continued to experience persistent oral ulceration, conjunctival injection, diffuse alopecia, and cutaneous lesions, although his arthralgia remained improved. Prednisolone 30 mg daily was prescribed with a planned taper of 5 mg every five days while awaiting the skin biopsy results.

Repeat laboratory evaluation demonstrated reactive rapid plasma reagin (RPR) with a titer of 1:256 and positive *T. pallidum* hemagglutination assay (TPHA) with a titer exceeding 1:10,240. Routine hematologic and biochemical investigations were largely within normal limits except for persistently low C4 (0.05 g/L), elevated ESR (48 mm/hour), mildly elevated glucose (6.1 mmol/L), reduced transferrin (2.1 g/L), and mildly increased red cell distribution width (RDW). Autoimmune testing again showed ANA positivity (1:160, homogeneous pattern) with negative anti-dsDNA antibodies. Extractable nuclear antigen antibodies were predominantly negative except for weak positivity of anti-Mi-2β, anti-RP11, and anti-sp100 antibodies. Antineutrophil cytoplasmic antibodies (MPO-ANCA and PR3-ANCA) were negative. HLA-B27 and HLA-B51 testing were also negative (Table 2).

**Table 2:** Follow-Up Laboratory Investigations During Diagnostic Evaluation Follow-up laboratory investigations obtained during the diagnostic evaluation after the initial assessment. Results include syphilis serology, complement studies, inflammatory markers, autoimmune serology, ANCA panel, and HLA typing. ANA: antinuclear antibody; anti-dsDNA: anti-double-stranded DNA antibody; anti-Mi-2β: anti-chromodomain helicase DNA-binding protein 4 (CHD4) antibody; anti-RP11: anti-RNA polymerase III subunit RP11 antibody; anti-sp100: anti-sp100 antibody; MPO-ANCA: myeloperoxidase antineutrophil cytoplasmic antibody; PR3-ANCA: proteinase 3 antineutrophil cytoplasmic antibody; HLA: human leukocyte antigen; RPR: rapid plasma reagin; TPHA: *Treponema pallidum* hemagglutination assay; ESR: erythrocyte sedimentation rate; CRP: C-reactive protein.

Investigation	Result	Reference range (Unit)
Syphilis Serology		
Rapid Plasma Reagin (RPR)	Reactive, titer 1:256	-
*Treponema pallidum* Hemagglutination Assay (TPHA)	Positive, titer >1:10,240	-
Complement Studies		
C3	0.93	0.79 - 1.52 (g/L)
C4	0.05	0.16 - 0.38 (g/L)
Inflammatory Markers		
ESR	48	0 - 15 (mm/hr)
CRP	6	0 - 8 (mg/L)
Autoimmune Serology		
ANA	Positive (1:160, homogeneous pattern)	-
Anti-dsDNA	Negative	-
Anti-Mi-2β	Weak Positive	-
Anti-RP11	Weak Positive	-
Anti-sp100	Weak Positive	-
ANCA Panel		
MPO-ANCA	Negative	-
PR3-ANCA	Negative	-
HLA Typing		
HLA-B27	Negative	-
HLA-B51	Negative	-

Histopathological examination of a punch biopsy specimen obtained from the right flank demonstrated mild epidermal atrophy with prominent vacuolar interface dermatitis. The superficial dermis showed a perivascular lymphoplasmacytic inflammatory infiltrate admixed with scattered interstitial neutrophils. Several neutrophils extended into the epidermis and were aligned along the dermoepidermal junction. Periodic acid-Schiff (PAS) staining demonstrated focal thickening of the basement membrane zone with areas of disruption, while Alcian blue staining showed no increase in dermal mucin deposition (Figure 3). The initial histopathological impression favored acute cutaneous lupus erythematosus (ACLE). However, after clinicopathological correlation and review of the newly available serologic findings demonstrating strongly positive syphilis serology, the pathologist recommended secondary syphilis as an important differential diagnosis while advising exclusion of an underlying connective tissue disease. 

**Figure 3:**
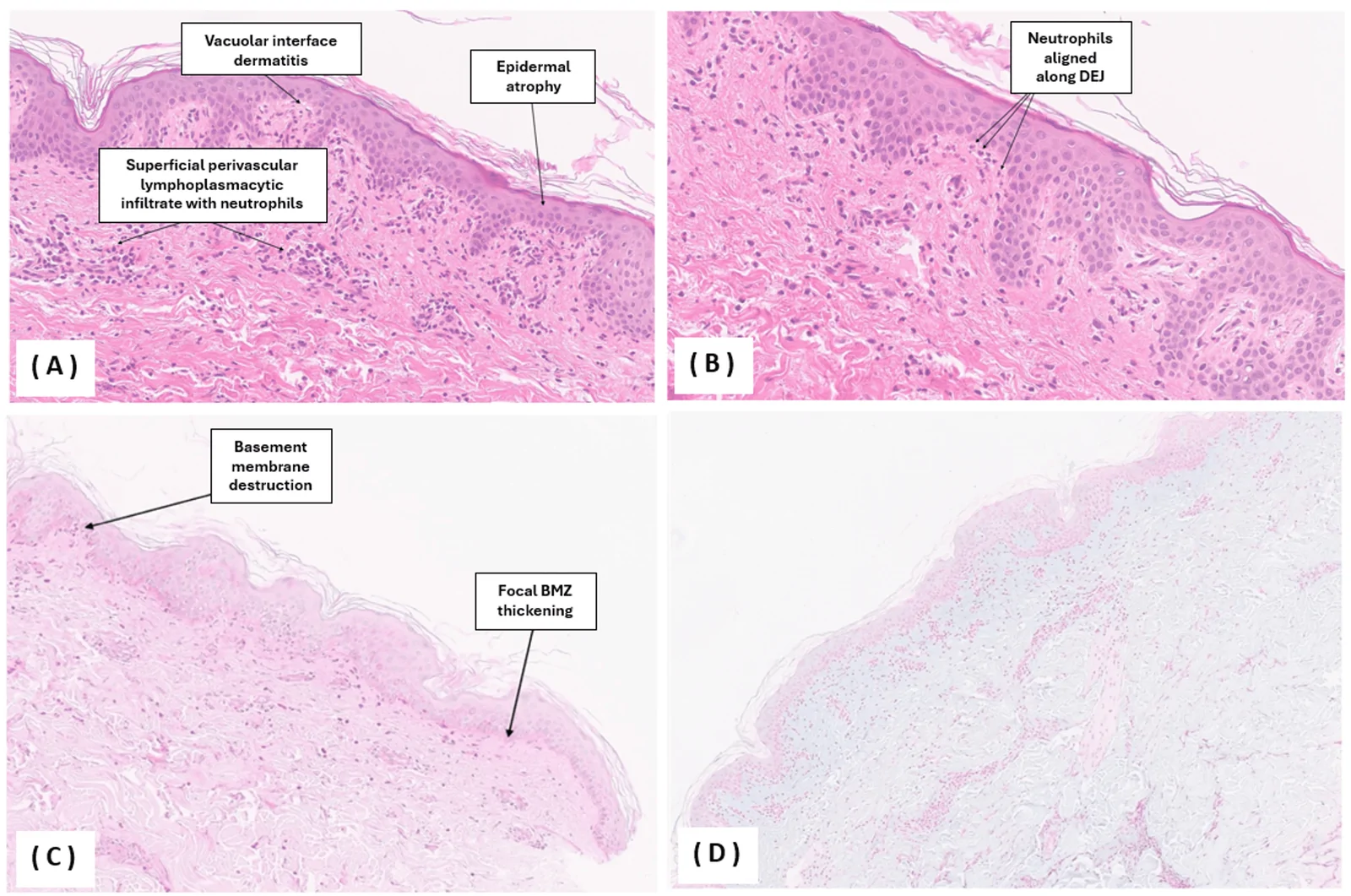
Histopathological Examination of the Punch Biopsy Specimen From the Right Flank (A) Hematoxylin and eosin (H&E) stain demonstrating mild epidermal atrophy, prominent vacuolar interface dermatitis, and a superficial perivascular lymphoplasmacytic inflammatory infiltrate admixed with scattered interstitial neutrophils. (B) Higher-power H&E view highlighting neutrophils aligned along the dermoepidermal junction (DEJ). (C) Periodic acid-Schiff (PAS) stain demonstrating focal thickening of the basement membrane zone (BMZ) with focal areas of basement membrane disruption. (D) Alcian blue stain demonstrating no significant increase in dermal mucin deposition.

Given the persistent bilateral conjunctival injection, the patient underwent ophthalmologic evaluation to assess for possible ocular involvement. Visual acuity was intact, and fundoscopic and macular examinations were within normal limits. Intraocular pressure was also within normal limits. No findings suggestive of ocular syphilis were identified on ophthalmologic assessment, and the ocular symptoms were ultimately attributed to dry eye disease. Following the diagnosis of secondary syphilis, hydroxychloroquine, methotrexate, and corticosteroid therapy were discontinued, and the patient was treated with benzathine penicillin G 2.4 million units intramuscularly once weekly for three consecutive weeks. At two-month follow-up, the generalized cutaneous eruption, oral ulceration, conjunctival redness, and polyarthralgia had resolved, with marked improvement of the diffuse alopecia. Repeat serologic testing demonstrated a decline in the RPR titer from 1:256 before treatment to 1:128 at two months (Table 3).

**Table 3:** Clinical Timeline From Presentation to Follow-Up Summary of the patient's clinical course from initial presentation through diagnosis, treatment, and follow-up. ANA: antinuclear antibody; SLE: systemic lupus erythematosus; ACLE: acute cutaneous lupus erythematosus; RPR: rapid plasma reagin; TPHA: *Treponema pallidum* hemagglutination assay; IM: intramuscular.

Clinical stage	Key findings/investigations	Management and outcome
Initial presentation	Conjunctival injection, polyarthralgia, generalized maculopapular eruption, alopecia, and oral ulcer; ANA 1:160, low C4, elevated inflammatory markers	SLE favored; hydroxychloroquine initiated
Follow-up	Persistent cutaneous and mucosal manifestations despite improvement in arthralgia	Methotrexate initiated, followed by a short course of prednisolone; skin biopsy obtained
Diagnostic reassessment	Biopsy initially favored ACLE; RPR 1:256 and TPHA >1:10,240; clinicopathologic correlation supported secondary syphilis	Diagnosis revised to secondary syphilis
Treatment	Secondary syphilis favored	Benzathine penicillin G 2.4 million units IM weekly for three weeks
Two-month follow-up	Resolution of presenting symptoms; RPR decreased from 1:256 to 1:128	Clinical improvement with declining RPR titer

## Discussion

Syphilis, caused by *T. pallidum* subspecies *pallidum*, remains a global health concern despite the availability of effective antimicrobial therapy. Secondary syphilis represents the hematogenous dissemination stage of infection and typically develops weeks to months after the primary chancre. During this phase, widespread dissemination of spirochetes results in highly variable mucocutaneous and systemic manifestations, earning syphilis the designation of the "Great Imitator." The clinical spectrum ranges from the classic papulosquamous eruption involving the trunk, extremities, palms, and soles to atypical presentations that mimic inflammatory, autoimmune, and neoplastic disorders, frequently delaying diagnosis. Constitutional symptoms, generalized lymphadenopathy, inflammatory arthralgia, oral mucosal lesions, and patchy "moth-eaten" alopecia further contribute to its diverse presentation [2].

Because of its protean manifestations, secondary syphilis is capable of mimicking numerous autoimmune diseases, particularly SLE. Several reports have described patients initially diagnosed with lupus based on clinical manifestations such as photosensitivity, alopecia, oral ulceration, arthritis, constitutional symptoms, and positive autoimmune serology before the diagnosis of secondary syphilis was ultimately established [3].

Our patient presented with inflammatory polyarthralgia, diffuse alopecia, oral ulceration, conjunctival injection, constitutional symptoms, and a generalized pruritic maculopapular eruption. Laboratory evaluation demonstrated ANA positivity (1:160) and hypocomplementemia, while the skin biopsy was initially interpreted as ACLE, collectively supporting an initial diagnosis of SLE. Such overlap illustrates one of the principal diagnostic challenges posed by secondary syphilis.

Although ANA positivity is commonly associated with connective tissue diseases, false-positive ANA results have been reported in chronic infections, including syphilis. Likewise, nonspecific inflammatory markers and complement abnormalities should be interpreted cautiously, particularly when the clinical picture contains atypical features [3]. In our patient, persistent ocular symptoms, significant weight loss, and night sweats were less characteristic of SLE and should prompt consideration of alternative infectious etiologies. Ultimately, strongly reactive nontreponemal and treponemal serologic tests redirected the diagnostic evaluation toward secondary syphilis.

Histopathologic examination remains an important diagnostic adjunct but should not be interpreted in isolation. Classical histopathologic features of ACLE include vacuolar interface dermatitis, basal keratinocyte degeneration, superficial and deep perivascular lymphocytic infiltrates, dermal mucin deposition, and basement membrane thickening [4]. However, secondary syphilis may demonstrate considerable histologic heterogeneity, including interface dermatitis, interstitial inflammation, endothelial swelling, acanthosis, psoriasiform epidermal hyperplasia, and perivascular inflammatory infiltrates, resulting in substantial overlap with connective tissue diseases [5].

In our patient, the biopsy demonstrated vacuolar interface dermatitis, perivascular and interstitial inflammatory infiltrates, neutrophils along the dermoepidermal junction, and focal basement membrane thickening on PAS staining. These findings initially favored ACLE. However, following review of the patient's markedly positive syphilis serology, the pathologist appropriately revised the interpretation to include secondary syphilis in the differential diagnosis. Recent clinicopathologic studies have demonstrated that plasma cells and endothelial swelling - traditionally regarded as characteristic features of secondary syphilis - may be absent in a substantial proportion of biopsies, emphasizing that no single histopathologic feature is pathognomonic. Instead, interpretation requires integration of clinical, serologic, and histologic findings [5].

This case highlights the indispensable role of clinicopathologic correlation in the diagnosis of atypical inflammatory dermatoses. Histopathologic findings alone suggested cutaneous lupus erythematosus, whereas serologic testing strongly supported secondary syphilis. Neither modality independently established the final diagnosis. Rather, communication between the treating clinicians and dermatopathologist prompted reinterpretation of the biopsy within the appropriate clinical context.

Secondary syphilis should therefore remain an important differential diagnosis in patients presenting with multisystem disease suggestive of connective tissue disorders, particularly when constitutional symptoms, unusual cutaneous findings, or incomplete fulfillment of classification criteria are present. Early recognition is essential because delayed diagnosis may expose patients to unnecessary immunosuppressive therapy while postponing curative antimicrobial treatment [6].

Several limitations should be acknowledged. *T. pallidum* immunohistochemistry and molecular testing were not performed on the skin biopsy; therefore, the presence of the organism within the cutaneous lesions was not directly demonstrated, and the diagnosis relied on clinicopathologic correlation with strongly positive syphilis serology and subsequent clinical response to antimicrobial therapy. In addition, concomitant SLE could not be definitively excluded. Although the patient demonstrated several lupus-like features, including ANA positivity, low C4 levels, oral ulceration, alopecia, arthralgia, and histopathologic findings initially favoring ACLE, the available data were insufficient to establish whether he fulfilled the 2019 EULAR/ACR SLE classification criteria. Furthermore, post-treatment ANA and complement levels were not available to determine whether these abnormalities normalized following treatment of syphilis. Nevertheless, resolution of the cutaneous, mucosal, articular, and ocular manifestations following antisyphilitic therapy, together with improvement in alopecia and a decline in RPR titer, favored secondary syphilis as the principal explanation for the clinical presentation. However, the possibility of concomitant SLE or infection-associated lupus-like immunologic abnormalities cannot be completely excluded.

Cases of secondary syphilis masquerading as SLE remain uncommon but are increasingly recognized in the literature. Previously reported patients have presented with malar eruptions, photosensitivity, arthritis, alopecia, lymphadenopathy, positive ANA, and histopathologic findings suggestive of lupus, often leading to initial treatment for autoimmune disease before syphilis serology established the correct diagnosis [7]. Our case is distinctive because multiple diagnostic domains - including clinical manifestations, autoimmune serology, complement abnormalities, and histopathology - simultaneously supported a diagnosis of SLE, creating a particularly convincing autoimmune phenotype. Recognition of the markedly positive RPR and TPHA results, together with clinicopathologic reassessment, prevented further diagnostic error and underscores the importance of maintaining a high index of suspicion for syphilis in patients with atypical lupus-like presentations.

## Conclusions

Secondary syphilis should remain an important diagnostic consideration in patients presenting with lupus-like mucocutaneous and systemic manifestations, even in the presence of positive autoimmune serology and histopathologic findings suggestive of cutaneous lupus erythematosus. This case underscores the importance of comprehensive clinicopathologic correlation and appropriate infectious disease screening before establishing a diagnosis of autoimmune disease or initiating long-term immunosuppressive therapy. However, the clinical response to antisyphilitic treatment favored secondary syphilis as the principal explanation for the presentation; concomitant SLE could not be definitively excluded. Recognizing this uncommon presentation can facilitate prompt treatment and prevent unnecessary morbidity associated with delayed diagnosis and inappropriate management.
